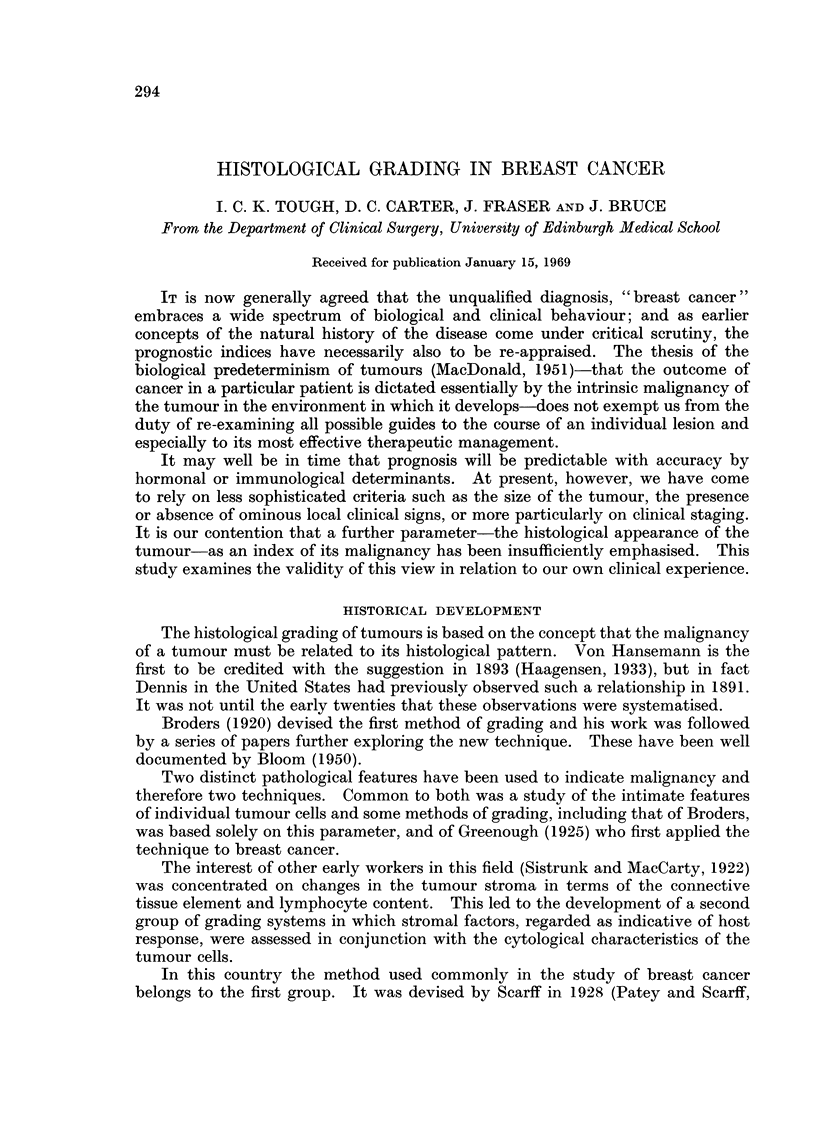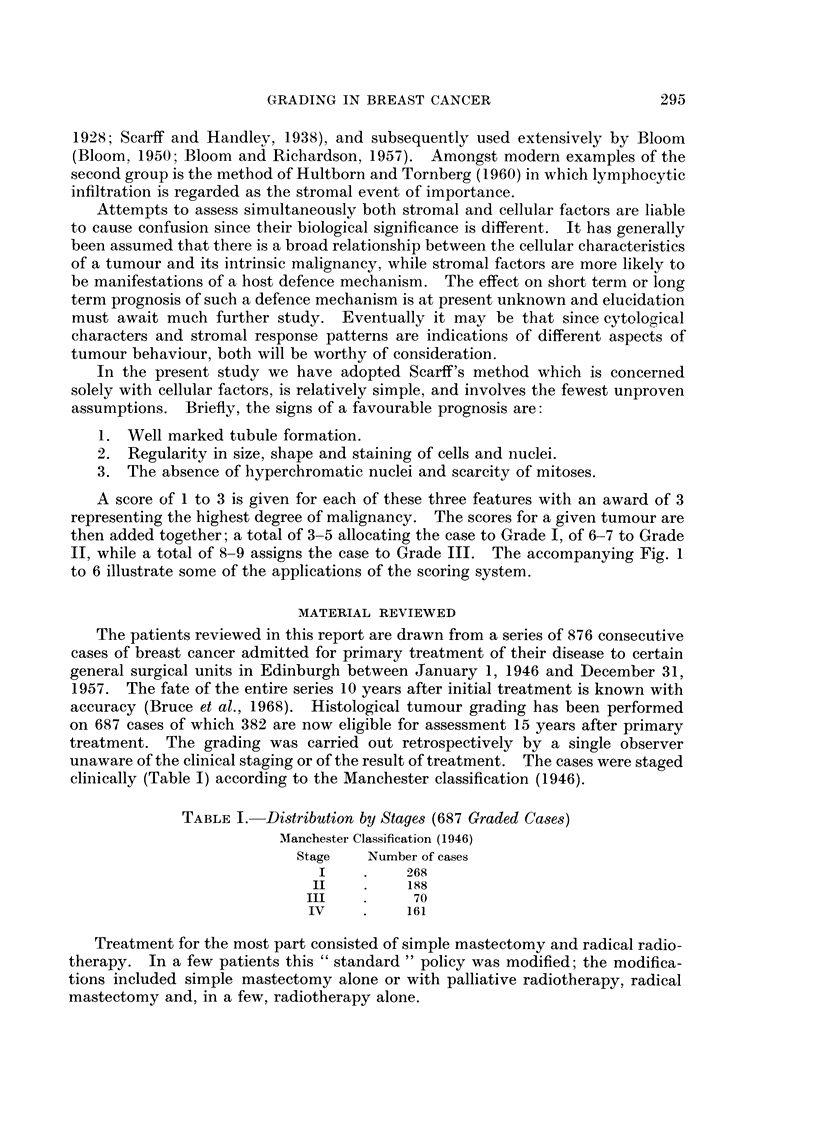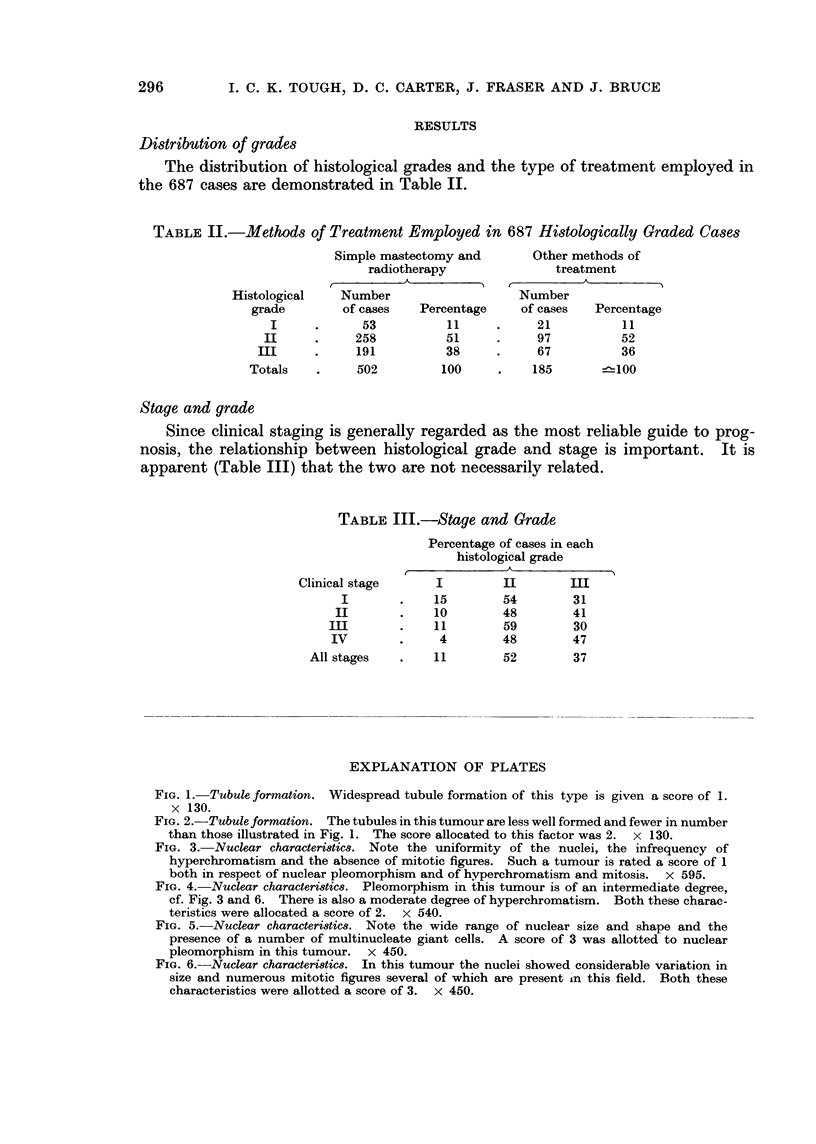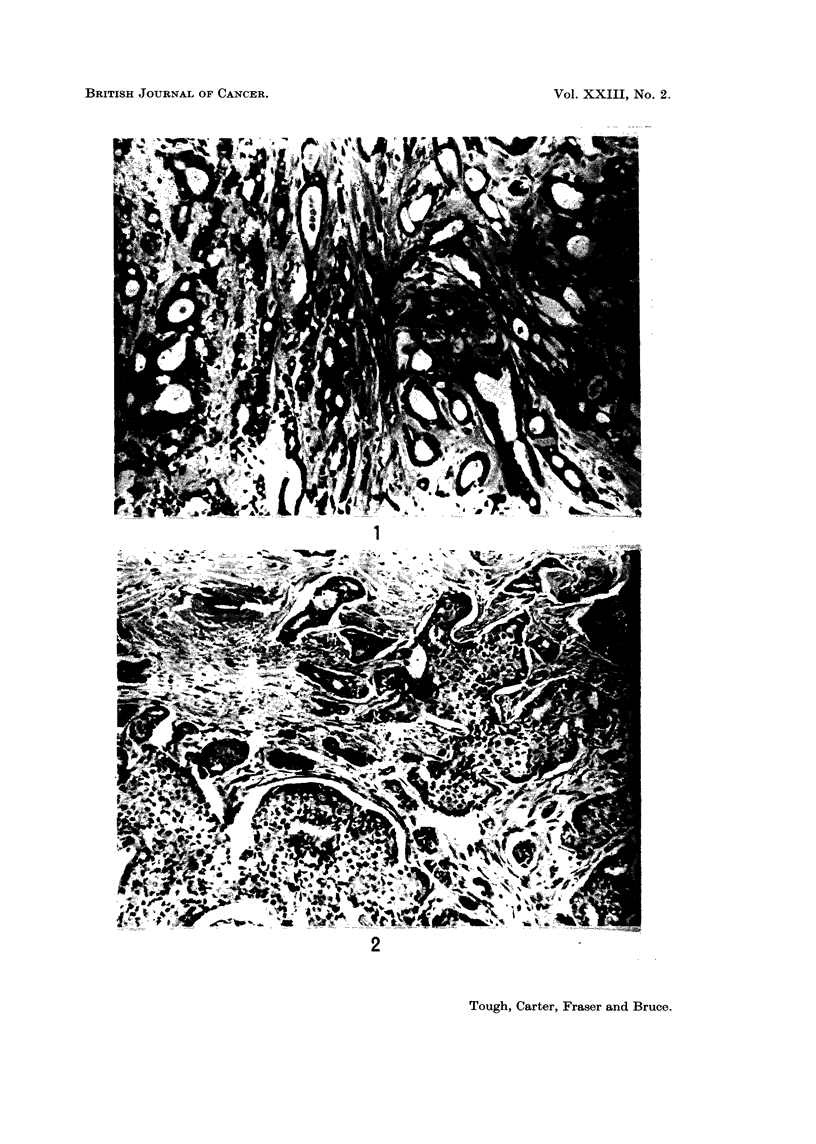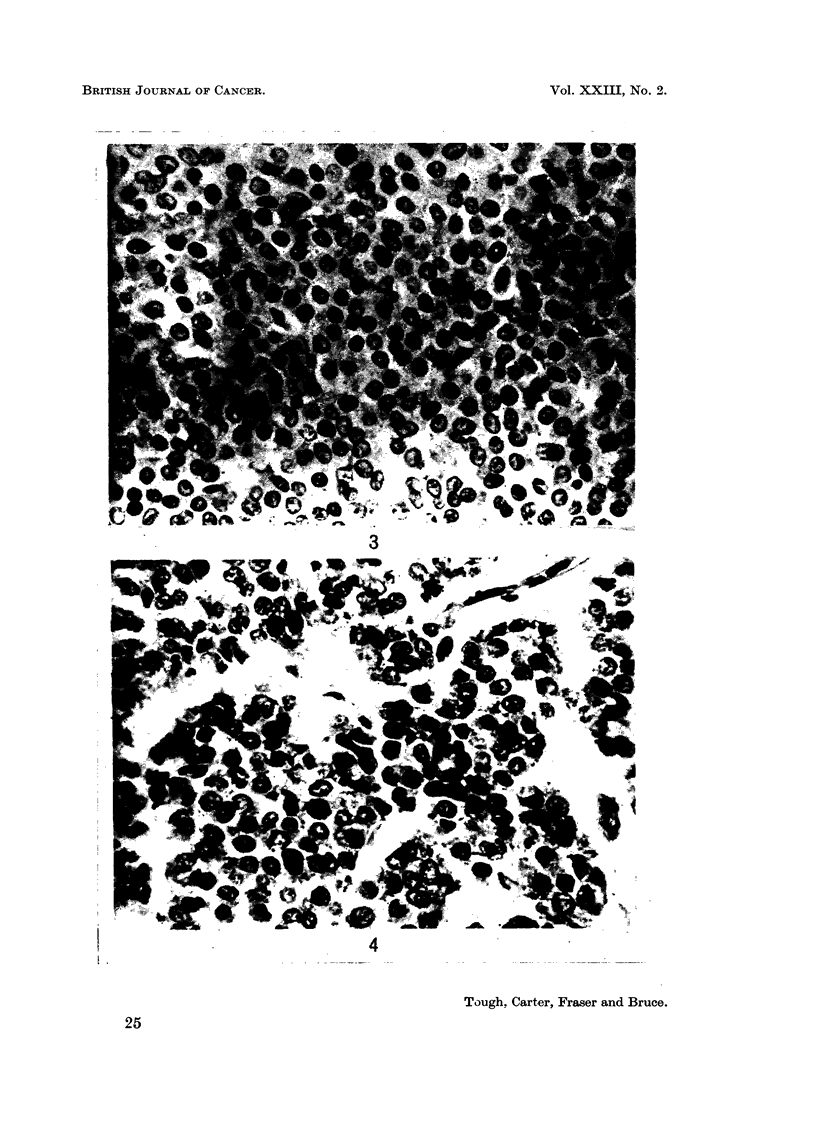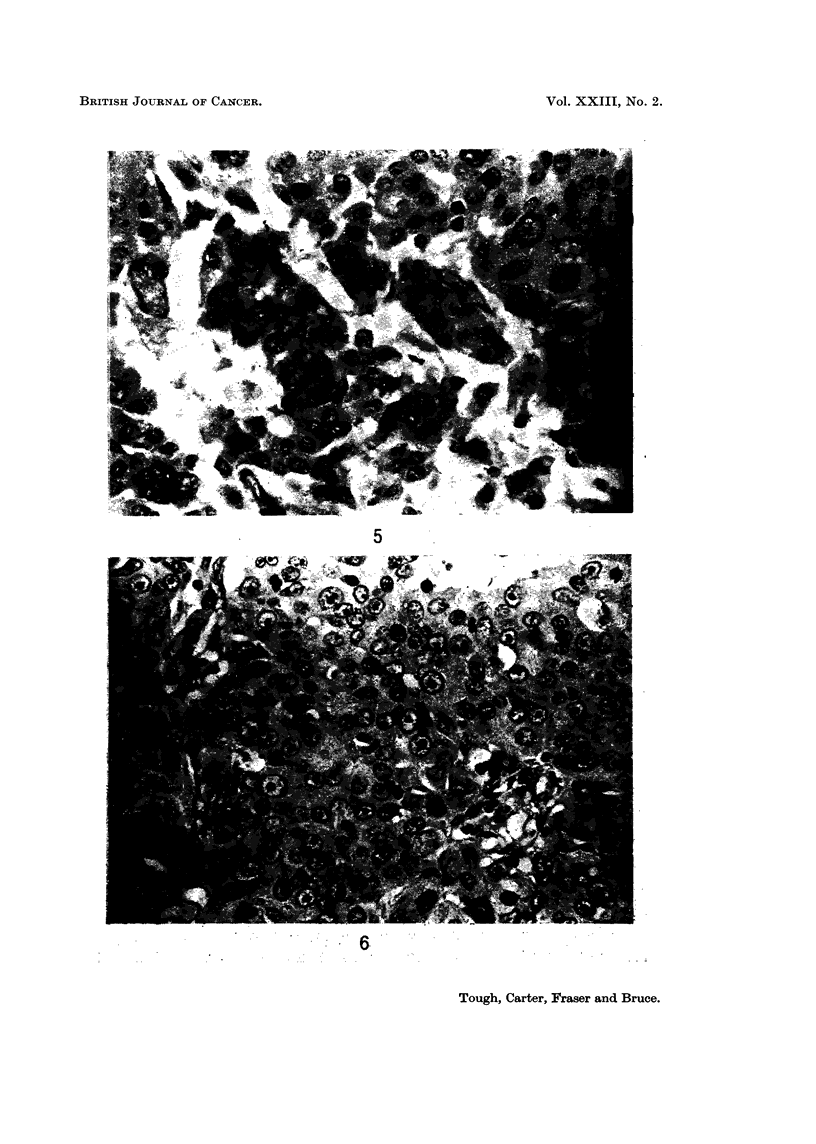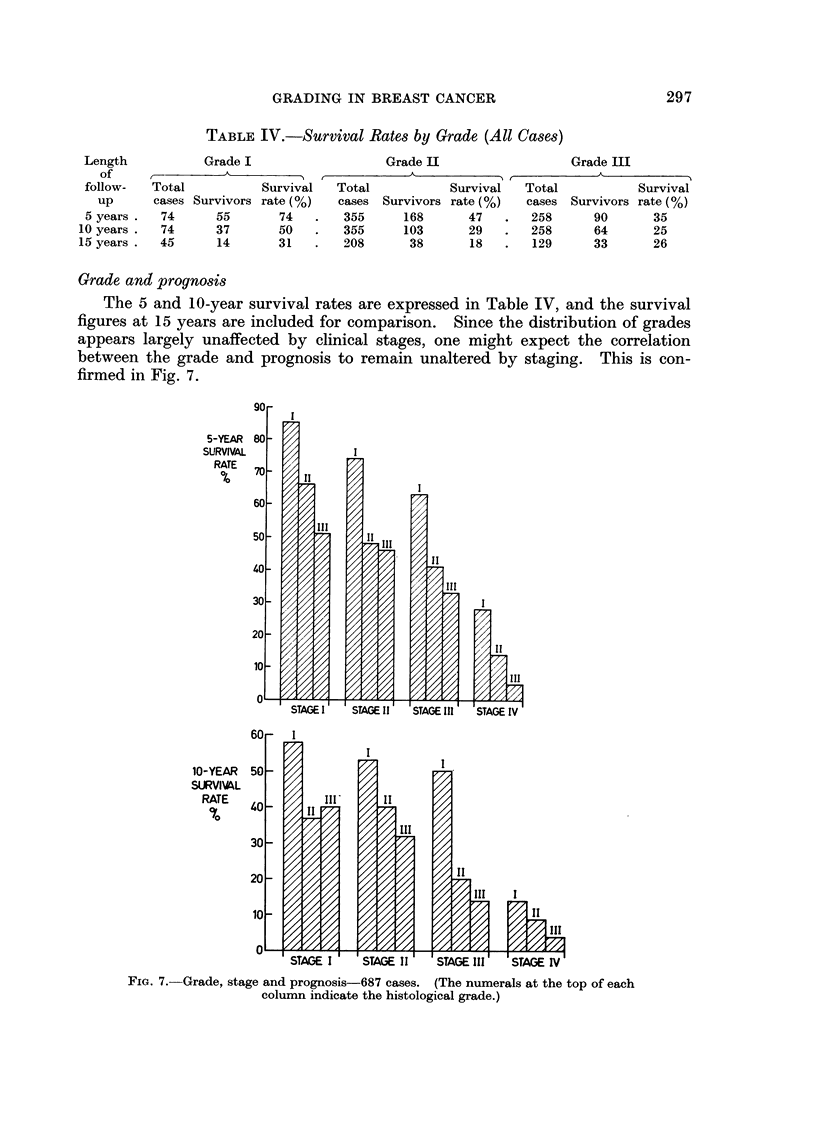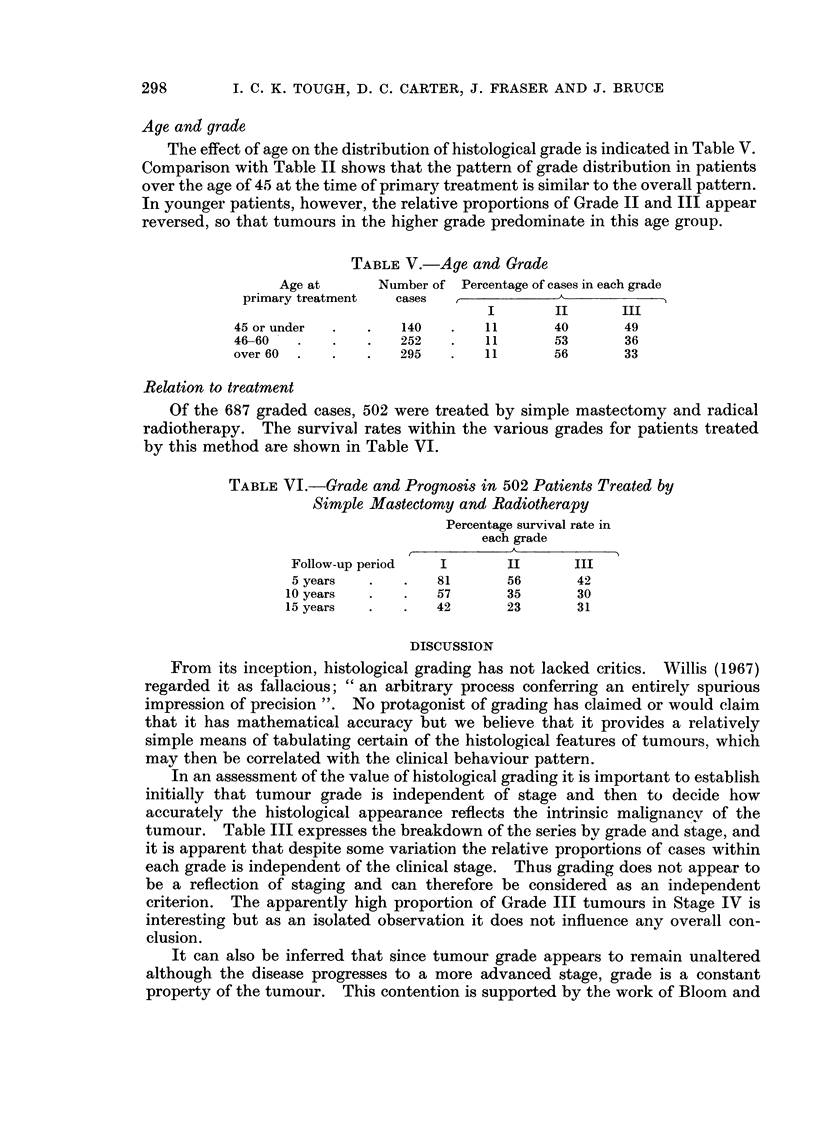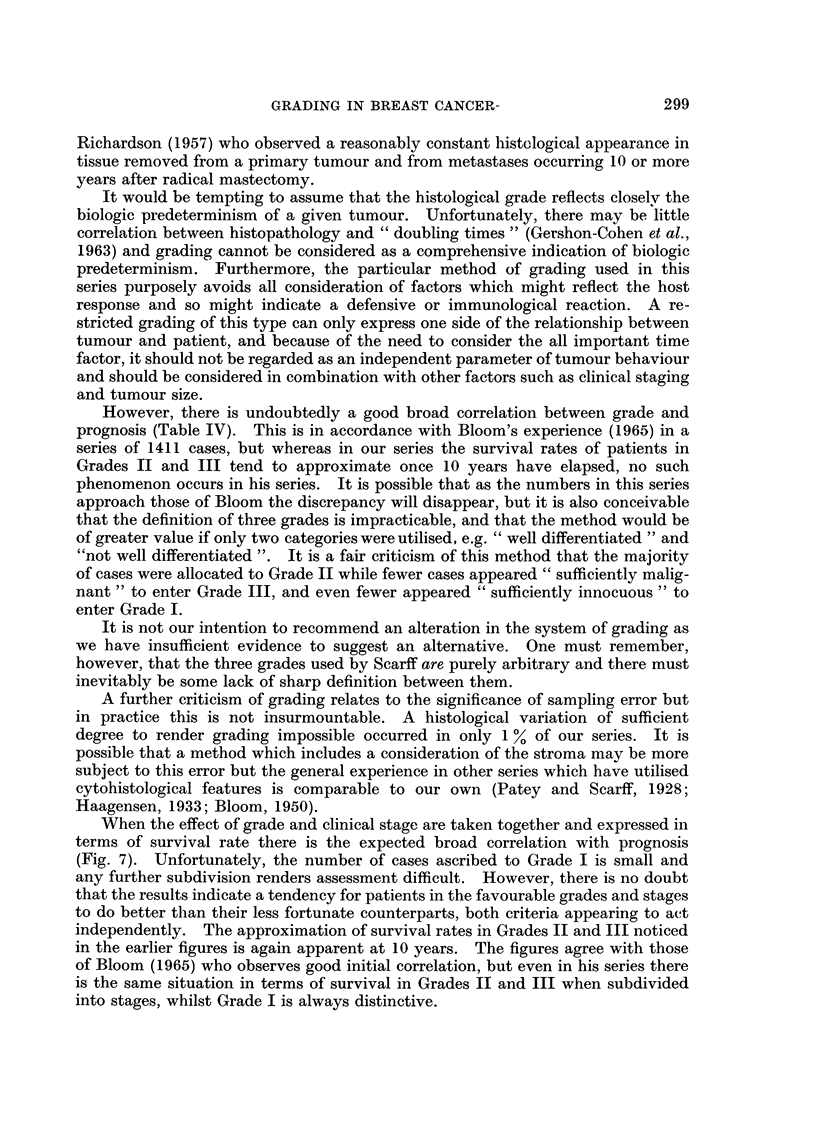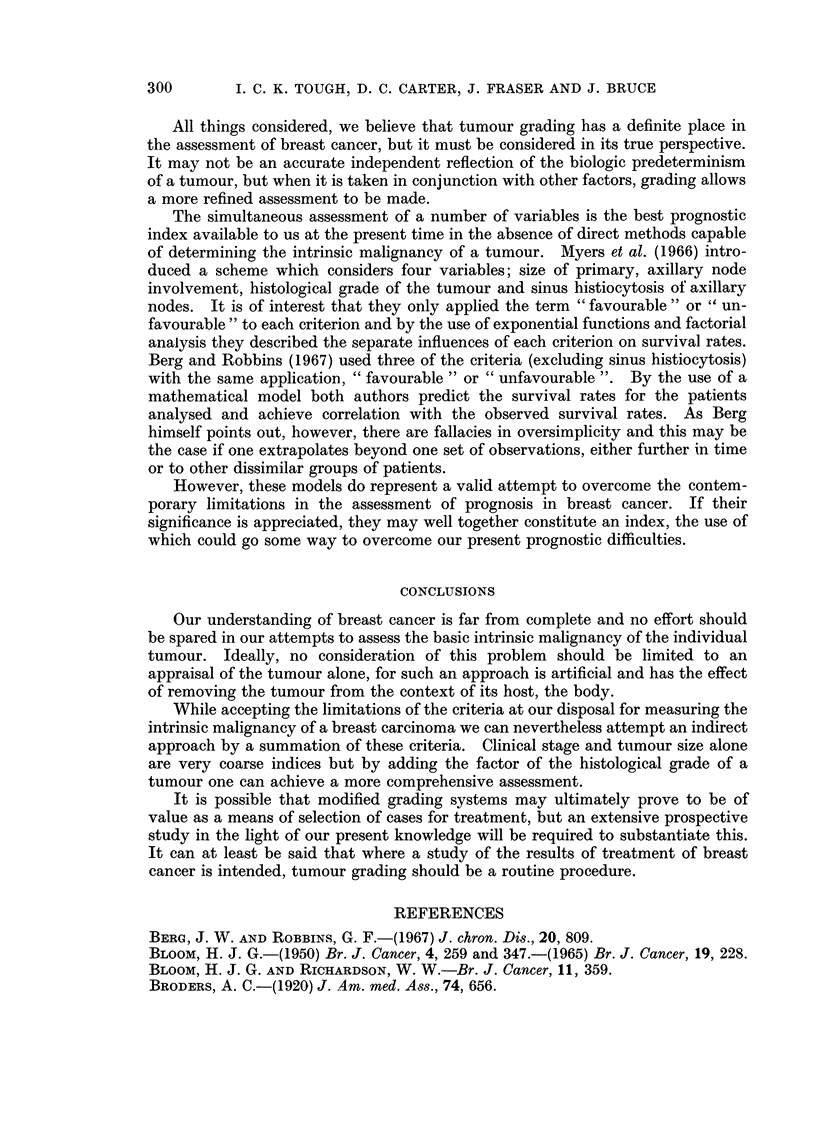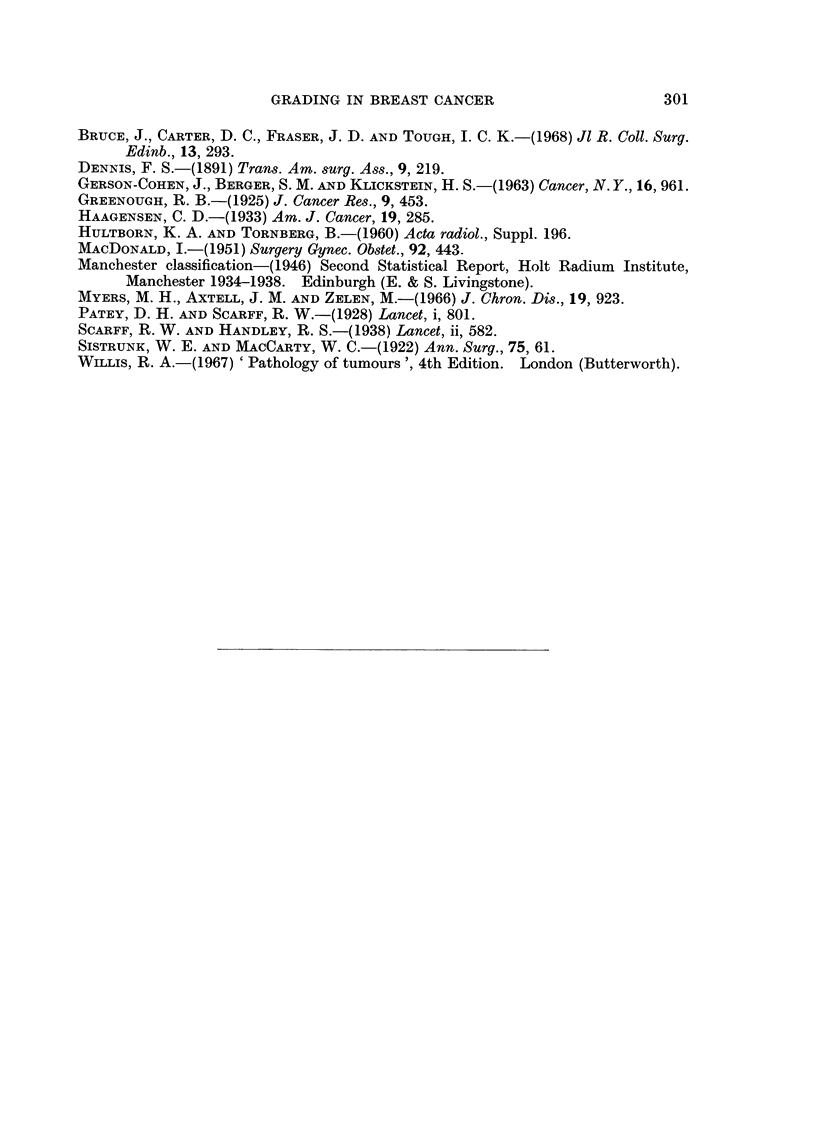# Histological grading in breast cancer.

**DOI:** 10.1038/bjc.1969.39

**Published:** 1969-06

**Authors:** I. C. Tough, D. C. Carter, J. Fraser, J. Bruce

## Abstract

**Images:**


### HISTOLOGICAL GRADING IN BREAST CANCER

I. C. K. TOUGH, D. C. CARTER, J. FRASER AND J. BRUCE
*From the Department of Clinical Surgery, University of Edinburgh Medical School*

Received for publication January 15, 1969

It is now generally agreed that the unqualified diagnosis, "breast cancer" embraces a wide spectrum of biological and clinical behaviour; and as earlier concepts of the natural history of the disease come under critical scrutiny, the prognostic indices have necessarily also to be re-appraised. The thesis of the biological predeterminism of tumours (MacDonald, 1951)—that the outcome of cancer in a particular patient is dictated essentially by the intrinsic malignancy of the tumour in the environment in which it develops—does not exempt us from the duty of re-examining all possible guides to the course of an individual lesion and especially to its most effective therapeutic management.

It may well be in time that prognosis will be predictable with accuracy by hormonal or immunological determinants. At present, however, we have come to rely on less sophisticated criteria such as the size of the tumour, the presence or absence of ominous local clinical signs, or more particularly on clinical staging. It is our contention that a further parameter—the histological appearance of the tumour—as an index of its malignancy has been insufficiently emphasised. This study examines the validity of this view in relation to our own clinical experience.

### HISTORICAL DEVELOPMENT

The histological grading of tumours is based on the concept that the malignancy of a tumour must be related to its histological pattern. Von Hansemann is the first to be credited with the suggestion in 1893 (Haagensen, 1933), but in fact Dennis in the United States had previously observed such a relationship in 1891. It was not until the early twenties that these observations were systematised.

Broders (1920) devised the first method of grading and his work was followed by a series of papers further exploring the new technique. These have been well documented by Bloom (1950).

Two distinct pathological features have been used to indicate malignancy and therefore two techniques. Common to both was a study of the intimate features of individual tumour cells and some methods of grading, including that of Broders, was based solely on this parameter, and of Greenough (1925) who first applied the technique to breast cancer.

The interest of other early workers in this field (Sistrunk and MacCarty, 1922) was concentrated on changes in the tumour stroma in terms of the connective tissue element and lymphocyte content. This led to the development of a second group of grading systems in which stromal factors, regarded as indicative of host response, were assessed in conjunction with the cytological characteristics of the tumour cells.

In this country the method used commonly in the study of breast cancer belongs to the first group. It was devised by Scarff in 1928 (Patey and Scarff,

1928; Scarff and Handley, 1938), and subsequently used extensively by Bloom (Bloom, 1950; Bloom and Richardson, 1957). Amongst modern examples of the second group is the method of Hultborn and Tornberg (1960) in which lymphocytic infiltration is regarded as the stromal event of importance.

Attempts to assess simultaneously both stromal and cellular factors are liable to cause confusion since their biological significance is different. It has generally been assumed that there is a broad relationship between the cellular characteristics of a tumour and its intrinsic malignancy, while stromal factors are more likely to be manifestations of a host defence mechanism. The effect on short term or long term prognosis of such a defence mechanism is at present unknown and elucidation must await much further study. Eventually it may be that since cytological characters and stromal response patterns are indications of different aspects of tumour behaviour, both will be worthy of consideration.

In the present study we have adopted Scarff's method which is concerned solely with cellular factors, is relatively simple, and involves the fewest unproven assumptions. Briefly, the signs of a favourable prognosis are:

1. Well marked tubule formation.
2. Regularity in size, shape and staining of cells and nuclei.
3. The absence of hyperchromatic nuclei and scarcity of mitoses.

A score of 1 to 3 is given for each of these three features with an award of 3 representing the highest degree of malignancy. The scores for a given tumour are then added together; a total of 3–5 allocating the case to Grade I, of 6–7 to Grade II, while a total of 8–9 assigns the case to Grade III. The accompanying Fig. 1 to 6 illustrate some of the applications of the scoring system.

### MATERIAL REVIEWED

The patients reviewed in this report are drawn from a series of 876 consecutive cases of breast cancer admitted for primary treatment of their disease to certain general surgical units in Edinburgh between January 1, 1946 and December 31, 1957. The fate of the entire series 10 years after initial treatment is known with accuracy (Bruce *et al.*, 1968). Histological tumour grading has been performed on 687 cases of which 382 are now eligible for assessment 15 years after primary treatment. The grading was carried out retrospectively by a single observer unaware of the clinical staging or of the result of treatment. The cases were staged clinically (Table I) according to the Manchester classification (1946).

TABLE I.—*Distribution by Stages* (687 *Graded Cases*)

Manchester Classification (1946)

| Stage | Number of cases |
|---|---|
| I | 268 |
| II | 188 |
| III | 70 |
| IV | 161 |

Treatment for the most part consisted of simple mastectomy and radical radiotherapy. In a few patients this " standard " policy was modified; the modifications included simple mastectomy alone or with palliative radiotherapy, radical mastectomy and, in a few, radiotherapy alone.

### RESULTS

*Distribution of grades*

The distribution of histological grades and the type of treatment employed in the 687 cases are demonstrated in Table II.

TABLE II.—*Methods of Treatment Employed in* 687 *Histologically Graded Cases*

| | Simple mastectomy and radiotherapy | | Other methods of treatment | |
|---|---|---|---|---|
| Histological grade | Number of cases | Percentage | Number of cases | Percentage |
| I | 53 | 11 | 21 | 11 |
| II | 258 | 51 | 97 | 52 |
| III | 191 | 38 | 67 | 36 |
| Totals | 502 | 100 | 185 | ≏100 |

*Stage and grade*

Since clinical staging is generally regarded as the most reliable guide to prognosis, the relationship between histological grade and stage is important. It is apparent (Table III) that the two are not necessarily related.

TABLE III.—*Stage and Grade*

| | Percentage of cases in each histological grade | | |
|---|---|---|---|
| Clinical stage | I | II | III |
| I | 15 | 54 | 31 |
| II | 10 | 48 | 41 |
| III | 11 | 59 | 30 |
| IV | 4 | 48 | 47 |
| All stages | 11 | 52 | 37 |

### EXPLANATION OF PLATES

FIG. 1.—*Tubule formation.* Widespread tubule formation of this type is given a score of 1. × 130.

FIG. 2.—*Tubule formation.* The tubules in this tumour are less well formed and fewer in number than those illustrated in Fig. 1. The score allocated to this factor was 2. × 130.

FIG. 3.—*Nuclear characteristics.* Note the uniformity of the nuclei, the infrequency of hyperchromatism and the absence of mitotic figures. Such a tumour is rated a score of 1 both in respect of nuclear pleomorphism and of hyperchromatism and mitosis. × 595.

FIG. 4.—*Nuclear characteristics.* Pleomorphism in this tumour is of an intermediate degree, cf. Fig. 3 and 6. There is also a moderate degree of hyperchromatism. Both these characteristics were allocated a score of 2. × 540.

FIG. 5.—*Nuclear characteristics.* Note the wide range of nuclear size and shape and the presence of a number of multinucleate giant cells. A score of 3 was allotted to nuclear pleomorphism in this tumour. × 450.

FIG. 6.—*Nuclear characteristics.* In this tumour the nuclei showed considerable variation in size and numerous mitotic figures several of which are present in this field. Both these characteristics were allotted a score of 3. × 450.

1

2

Tough, Carter, Fraser and Bruce.

3

4

Tough, Carter, Fraser and Bruce.

5

6

Tough, Carter, Fraser and Bruce.

TABLE IV.—*Survival Rates by Grade (All Cases)*

| Length of follow-up | Grade I | | | Grade II | | | Grade III | | |
|---|---|---|---|---|---|---|---|---|---|
| | Total cases | Survivors | Survival rate (%) | Total cases | Survivors | Survival rate (%) | Total cases | Survivors | Survival rate (%) |
| 5 years . | 74 | 55 | 74 . | 355 | 168 | 47 . | 258 | 90 | 35 |
| 10 years . | 74 | 37 | 50 . | 355 | 103 | 29 . | 258 | 64 | 25 |
| 15 years . | 45 | 14 | 31 . | 208 | 38 | 18 . | 129 | 33 | 26 |

*Grade and prognosis*

The 5 and 10-year survival rates are expressed in Table IV, and the survival figures at 15 years are included for comparison. Since the distribution of grades appears largely unaffected by clinical stages, one might expect the correlation between the grade and prognosis to remain unaltered by staging. This is confirmed in Fig. 7.

FIG. 7.—Grade, stage and prognosis—687 cases. (The numerals at the top of each column indicate the histological grade.)

*Age and grade*

The effect of age on the distribution of histological grade is indicated in Table V. Comparison with Table II shows that the pattern of grade distribution in patients over the age of 45 at the time of primary treatment is similar to the overall pattern. In younger patients, however, the relative proportions of Grade II and III appear reversed, so that tumours in the higher grade predominate in this age group.

TABLE V.—*Age and Grade*

| Age at primary treatment | Number of cases | Percentage of cases in each grade | | |
|---|---|---|---|---|
| | | I | II | III |
| 45 or under | 140 | 11 | 40 | 49 |
| 46–60 | 252 | 11 | 53 | 36 |
| over 60 | 295 | 11 | 56 | 33 |

*Relation to treatment*

Of the 687 graded cases, 502 were treated by simple mastectomy and radical radiotherapy. The survival rates within the various grades for patients treated by this method are shown in Table VI.

TABLE VI.—*Grade and Prognosis in* 502 *Patients Treated by Simple Mastectomy and Radiotherapy*

| | Percentage survival rate in each grade | | |
|---|---|---|---|
| Follow-up period | I | II | III |
| 5 years | 81 | 56 | 42 |
| 10 years | 57 | 35 | 30 |
| 15 years | 42 | 23 | 31 |

DISCUSSION

From its inception, histological grading has not lacked critics. Willis (1967) regarded it as fallacious; " an arbitrary process conferring an entirely spurious impression of precision ". No protagonist of grading has claimed or would claim that it has mathematical accuracy but we believe that it provides a relatively simple means of tabulating certain of the histological features of tumours, which may then be correlated with the clinical behaviour pattern.

In an assessment of the value of histological grading it is important to establish initially that tumour grade is independent of stage and then to decide how accurately the histological appearance reflects the intrinsic malignancy of the tumour. Table III expresses the breakdown of the series by grade and stage, and it is apparent that despite some variation the relative proportions of cases within each grade is independent of the clinical stage. Thus grading does not appear to be a reflection of staging and can therefore be considered as an independent criterion. The apparently high proportion of Grade III tumours in Stage IV is interesting but as an isolated observation it does not influence any overall conclusion.

It can also be inferred that since tumour grade appears to remain unaltered although the disease progresses to a more advanced stage, grade is a constant property of the tumour. This contention is supported by the work of Bloom and

Richardson (1957) who observed a reasonably constant histological appearance in tissue removed from a primary tumour and from metastases occurring 10 or more years after radical mastectomy.

It would be tempting to assume that the histological grade reflects closely the biologic predeterminism of a given tumour. Unfortunately, there may be little correlation between histopathology and " doubling times " (Gershon-Cohen *et al.*, 1963) and grading cannot be considered as a comprehensive indication of biologic predeterminism. Furthermore, the particular method of grading used in this series purposely avoids all consideration of factors which might reflect the host response and so might indicate a defensive or immunological reaction. A restricted grading of this type can only express one side of the relationship between tumour and patient, and because of the need to consider the all important time factor, it should not be regarded as an independent parameter of tumour behaviour and should be considered in combination with other factors such as clinical staging and tumour size.

However, there is undoubtedly a good broad correlation between grade and prognosis (Table IV). This is in accordance with Bloom's experience (1965) in a series of 1411 cases, but whereas in our series the survival rates of patients in Grades II and III tend to approximate once 10 years have elapsed, no such phenomenon occurs in his series. It is possible that as the numbers in this series approach those of Bloom the discrepancy will disappear, but it is also conceivable that the definition of three grades is impracticable, and that the method would be of greater value if only two categories were utilised, e.g. " well differentiated " and "not well differentiated ". It is a fair criticism of this method that the majority of cases were allocated to Grade II while fewer cases appeared " sufficiently malignant " to enter Grade III, and even fewer appeared " sufficiently innocuous " to enter Grade I.

It is not our intention to recommend an alteration in the system of grading as we have insufficient evidence to suggest an alternative. One must remember, however, that the three grades used by Scarff *are* purely arbitrary and there must inevitably be some lack of sharp definition between them.

A further criticism of grading relates to the significance of sampling error but in practice this is not insurmountable. A histological variation of sufficient degree to render grading impossible occurred in only 1% of our series. It is possible that a method which includes a consideration of the stroma may be more subject to this error but the general experience in other series which have utilised cytohistological features is comparable to our own (Patey and Scarff, 1928; Haagensen, 1933; Bloom, 1950).

When the effect of grade and clinical stage are taken together and expressed in terms of survival rate there is the expected broad correlation with prognosis (Fig. 7). Unfortunately, the number of cases ascribed to Grade I is small and any further subdivision renders assessment difficult. However, there is no doubt that the results indicate a tendency for patients in the favourable grades and stages to do better than their less fortunate counterparts, both criteria appearing to act independently. The approximation of survival rates in Grades II and III noticed in the earlier figures is again apparent at 10 years. The figures agree with those of Bloom (1965) who observes good initial correlation, but even in his series there is the same situation in terms of survival in Grades II and III when subdivided into stages, whilst Grade I is always distinctive.

All things considered, we believe that tumour grading has a definite place in the assessment of breast cancer, but it must be considered in its true perspective. It may not be an accurate independent reflection of the biologic predeterminism of a tumour, but when it is taken in conjunction with other factors, grading allows a more refined assessment to be made.

The simultaneous assessment of a number of variables is the best prognostic index available to us at the present time in the absence of direct methods capable of determining the intrinsic malignancy of a tumour. Myers *et al.* (1966) introduced a scheme which considers four variables; size of primary, axillary node involvement, histological grade of the tumour and sinus histiocytosis of axillary nodes. It is of interest that they only applied the term "favourable" or "unfavourable" to each criterion and by the use of exponential functions and factorial analysis they described the separate influences of each criterion on survival rates. Berg and Robbins (1967) used three of the criteria (excluding sinus histiocytosis) with the same application, "favourable" or "unfavourable". By the use of a mathematical model both authors predict the survival rates for the patients analysed and achieve correlation with the observed survival rates. As Berg himself points out, however, there are fallacies in oversimplicity and this may be the case if one extrapolates beyond one set of observations, either further in time or to other dissimilar groups of patients.

However, these models do represent a valid attempt to overcome the contemporary limitations in the assessment of prognosis in breast cancer. If their significance is appreciated, they may well together constitute an index, the use of which could go some way to overcome our present prognostic difficulties.

### CONCLUSIONS

Our understanding of breast cancer is far from complete and no effort should be spared in our attempts to assess the basic intrinsic malignancy of the individual tumour. Ideally, no consideration of this problem should be limited to an appraisal of the tumour alone, for such an approach is artificial and has the effect of removing the tumour from the context of its host, the body.

While accepting the limitations of the criteria at our disposal for measuring the intrinsic malignancy of a breast carcinoma we can nevertheless attempt an indirect approach by a summation of these criteria. Clinical stage and tumour size alone are very coarse indices but by adding the factor of the histological grade of a tumour one can achieve a more comprehensive assessment.

It is possible that modified grading systems may ultimately prove to be of value as a means of selection of cases for treatment, but an extensive prospective study in the light of our present knowledge will be required to substantiate this. It can at least be said that where a study of the results of treatment of breast cancer is intended, tumour grading should be a routine procedure.

### REFERENCES

Berg, J. W. and Robbins, G. F.—(1967) *J. chron. Dis.*, **20**, 809.
Bloom, H. J. G.—(1950) *Br. J. Cancer*, **4**, 259 and 347.—(1965) *Br. J. Cancer*, **19**, 228.
Bloom, H. J. G. and Richardson, W. W.—*Br. J. Cancer*, **11**, 359.
Broders, A. C.—(1920) *J. Am. med. Ass.*, **74**, 656.

Bruce, J., Carter, D. C., Fraser, J. D. and Tough, I. C. K.—(1968) *Jl R. Coll. Surg. Edinb.*, **13**, 293.
Dennis, F. S.—(1891) *Trans. Am. surg. Ass.*, **9**, 219.
Gerson-Cohen, J., Berger, S. M. and Klickstein, H. S.—(1963) *Cancer, N.Y.*, **16**, 961.
Greenough, R. B.—(1925) *J. Cancer Res.*, **9**, 453.
Haagensen, C. D.—(1933) *Am. J. Cancer*, **19**, 285.
Hultborn, K. A. and Tornberg, B.—(1960) *Acta radiol.*, Suppl. 196.
MacDonald, I.—(1951) *Surgery Gynec. Obstet.*, **92**, 443.
Manchester classification—(1946) Second Statistical Report, Holt Radium Institute, Manchester 1934–1938. Edinburgh (E. & S. Livingstone).
Myers, M. H., Axtell, J. M. and Zelen, M.—(1966) *J. Chron. Dis.*, **19**, 923.
Patey, D. H. and Scarff, R. W.—(1928) *Lancet*, i, 801.
Scarff, R. W. and Handley, R. S.—(1938) *Lancet*, ii, 582.
Sistrunk, W. E. and MacCarty, W. C.—(1922) *Ann. Surg.*, **75**, 61.
Willis, R. A.—(1967) ' Pathology of tumours ', 4th Edition. London (Butterworth).